# 2086. PrEP Knowledge and Use among Reproductive Age Women in Miami, Florida

**DOI:** 10.1093/ofid/ofac492.1708

**Published:** 2022-12-15

**Authors:** Nicholas Fonseca Nogueira, Nicole Luisi, Ana S Salazar Zetina, Patricia Raccamarich, Nichole R Klatt, Deborah L Jones, Maria L L Alcaide

**Affiliations:** Division of Infectious Diseases, Department of Medicine, University of Miami Miller School of Medicine, Parkland, Florida; Emory University, Atlanta, Georgia; University of Miami, Miami, Florida; Division of Infectious Diseases, Department of Medicine, University of Miami Miller School of Medicine, Parkland, Florida; University of Minnesota Medical School, Minneapolis, Minnesota; University of Miami, Miami, Florida; Division of Infectious Diseases, Department of Medicine, University of Miami Miller School of Medicine, Parkland, Florida

## Abstract

**Background:**

Miami, FL is the epicenter of the HIV epidemic in the US, with 20% of new HIV infections occurring in women. Despite the effectiveness of PrEP, only 10% of eligible women benefit from its use. This study evaluates PrEP awareness and use, and factors associated with PrEP awareness among reproductive age sexually active women in Miami.

**Methods:**

From 2018 to 2022, cis-gender, HIV-negative, 18-45-year-old, sexually active women were recruited as part of a study evaluating HIV risk. Participants completed questionnaires assessing socio-demographics, HIV risk behaviors, prior HIV testing, PrEP awareness and use, and were tested for STIs (chlamydia, gonorrhea, trichomoniasis) and bacterial vaginosis (BV). Factors associated with PrEP awareness were analyzed by Chi-square, Fisher’s exact test, or studentized t-test. Multivariable logistic regression (MLR) was performed to identify factors associated with PrEP awareness.

**Results:**

295 women were enrolled; median age 31 (24-38) years, 49% Black, 39% White, 34% Hispanic, 46% income below poverty line, 24% consistent condom use, 1.6 (±2.57) mean number of partners in the previous month, 80% heterosexual, 73% previously tested for HIV, 22% STI, and 50% BV. Of the 63% who knew about PrEP, only 5% were on PrEP. Women aware of PrEP were more likely to be Black, non-Hispanic, have income below poverty line, non-heterosexual, report prior HIV testing, and have a STI or BV (all p< 0.05). Condom use and number of partners were not associated with PrEP awareness. In MLR, women with income below poverty line (OR=2.00 [1.04,3.87]; p=0.04), more sexual partners (OR=1.30 [1.01,1.68]; p=0.04), prior HIV testing (OR=6.42 [2.83,14.52]; p< 0.01), and BV (OR=2.28 [1.18,4.40]; p=0.01) were more likely to be aware of PrEP. Lower odds of PrEP awareness were associated with being Black (OR=0.38 [0.15,0.96]; p=0.04), Hispanic (OR=0.18 [0.08,0.39]; p< 0.01), Heterosexual (OR=0.29 [0.11,0.77]; p< 0.01), and reporting occasional condom use (OR=0.21 [0.08,0.56]; p< 0.01).

**Conclusion:**

Despite broad PrEP awareness, PrEP use is alarming low among those women at greatest risk. HIV testing presents an opportunity to increase PrEP awareness, but interventions to increase PrEP uptake are needed to end the HIV epidemic among women.

**Disclosures:**

**Maria L L. Alcaide, MD**, Gilead: Advisor/Consultant.

